# Oncogenic mechanisms of Lin28 in breast cancer: new functions and therapeutic opportunities

**DOI:** 10.18632/oncotarget.14891

**Published:** 2017-01-29

**Authors:** Hanchu Xiong, Wenhe Zhao, Ji Wang, Benjamin J. Seifer, Chenyang Ye, Yongxia Chen, Yunlu Jia, Cong Chen, Jianguo Shen, Linbo Wang, Xinbing Sui, Jichun Zhou

**Affiliations:** ^1^ Department of Surgical Oncology, Sir Run Run Shaw Hospital, Zhejiang University, Hangzhou, Zhejiang, China; ^2^ Biomedical Research Center and Key Laboratory of Biotherapy of Zhejiang Province, Hangzhou, Zhejiang, China; ^3^ USF Health Morsani College of Medicine, Tampa, FL, USA; ^4^ Cancer Institute (Key Laboratory of Cancer Prevention & Intervention, National Ministry of Education), Second Affiliated Hospital, Zhejiang University, Hangzhou, Zhejiang, China; ^5^ Department of Medical Oncology, Sir Run Run Shaw Hospital, Zhejiang University, Hangzhou, Zhejiang, China

**Keywords:** Lin28, Let-7, breast cancer, metastasis, drug resistance

## Abstract

The RNA binding protein Lin28 is best known for the critical role in cell development, recent researches also have implied its oncogenic function in various human cancers, including breast cancer. Specifically, aberrant Lin28 participates in multiple pathological processes, such as proliferation, metastasis, radiotherapy and chemotherapy resistance, metabolism, immunity and inflammation as well as stemness. In this review, we summarize the let-7-dependent and let-7-independent mechanism regulated by Lin28, focusing on its relation with tumor hallmarks in breast cancer, and subsequently discuss our present knowledge of Lin28 to develop a molecular-based therapeutic strategy against breast cancer.

## INTRODUCTION

Breast cancer is a complex and poorly understood disease which affects 1 in 10 females. It is the leading cause of death in women between the ages of 40 and 50 years [1]. Since breast cancer is highly heterogeneous in terms of biological behavior, researchers have made extensive efforts to conquer this challenge. In the last few decades, several novel molecular or cellular factors were identified in breast cancer, including Lin28 and its related factors [2]. Currently, the mainstream opinion is that Lin28 mainly functions as an oncogene. Mechanistically, Lin28 is involved in various pathological processes of cancers *via* let-7 dependent and let-7 independent pathways [3]. With a deeper understanding of how Lin28 is involved in breast cancer initiation and progression, new findings will be valuable in helping us to explore variably targetable mechanisms of breast cancer. The purpose of this article is to review existing literatures on the molecular mechanisms of Lin28, along with its roles in breast cancer. To our knowledge, this is the first review focusing on Lin28 in breast cancer. We believe that a more comprehensive understanding of Lin28 function will yield preventive, diagnostic, predictive and therapeutic advances for breast cancer.

## MOLECULAR MECHANISMS OF LIN28 AND ITS TWO PATHWAYS

### Basic knowledge of Lin28

**Figure 1:**
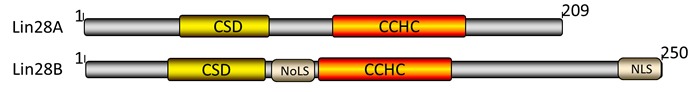
Domains of human Lin28A and Lin28B proteins Lin28A and Lin28B share several common domains: the CSD (yellow) and CCHC (orange), while Lin28B contains both NoLS and NLS (gray). Numbers denote amino acids.

Firstly identified as a regulator of developmental timing in Caenorhabditis elegans [4], Lin28 is now well established as an RNA binding protein and transcription factor implicated in stem cell differentiation, normal development, glucose metabolism, and cancer [5]. Lin28 has two RNA-binding motifs: a cold shock domain (CSD) and a Cys-Cys-His-Cys (CCHC) zinc finger domain [6]. Mammals produce two Lin28 paralogs: Lin28A and Lin28B, which have identical regions in structures and functional similarities but differ in a few respects [7]. For instance, Lin28B has additional sections: a nuclear localization signal (NLS) and a nucleolar localization signal (NoLS), and is therefore primarily located in nucleus and nucleolus. Lin28A on the other hand is predominantly located in the cytosol of cells (Figure 1).

The let-7 family of microRNA (miRNA), which was also firstly discovered in Caenorhabditis elegans [8], has been reported to be a key suppressive target of Lin28, and serves as a potent tumor suppressor *via* post-transcriptional repression of multiple oncogenic messenger RNA (mRNA) [9]. Meanwhile, the convergence of abundant *in vitro* and *in vivo* structural studies have discovered the molecular basis of the interaction between Lin28 and other target RNAs, whose Lin28 binding sites vary depending on their sequence and context [10]. It is therefore appropriate to assign the critical functions of Lin28 to one of two classifications: let-7 dependent and let-7 independent.

### Let-7 dependent functionality

**Figure 2:**
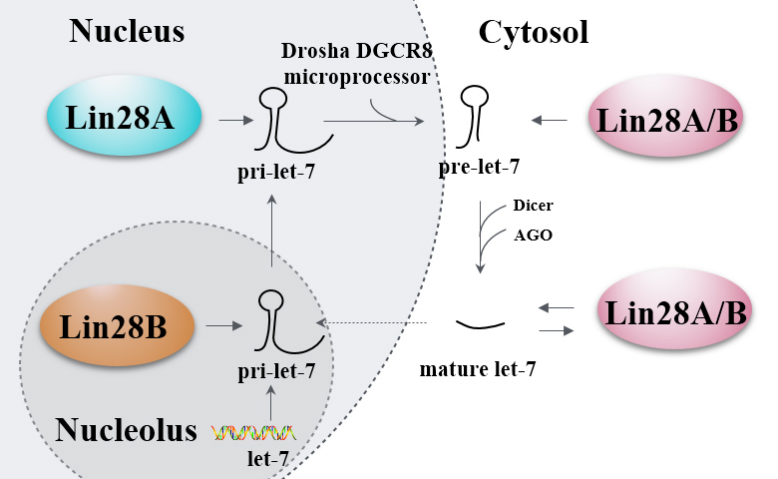
Lin28′s let-7-dependent functionality A primary let-7 (pri-let-7) transcript produced by let-7 gene is processed by the Drosha DGCR8 microprocessor in the nucleus. Then the generated precursor let-7 (pre-let-7) is transported to the cytosol and further processed by the Dicer and Argonaute proteins (AGO) to generate the mature let-7. The biogenesis of pri-let-7 is blocked by Lin28A in the nucleus and Lin28B in the nucleolus, the biogenesis of pre-let-7 and mature let-7 are blocked by Lin28A/B in the cytosol, and the mature let-7 can in turn block the biogenesis of Lin28A/B. Solid line arrows refer to induction and promotion, dash line arrows refer to only promotion, while hammerheads refer to inhibition.

Lin28 homologs (Lin28A and Lin28B) are small (< 30kDa) proteins which can block the processing of let-7 family members by binding to the terminal loop of the let-7 precursor (pre-let-7) hairpin *via* a CSD and two retroviral-like CHCC zinc-finger knuckles [11–13]. The GGAG sequences in the terminal loop of let-7 precursors serve as the binding sites for the zinc finger domains critical for let-7 regulation [14]. Subsequent reports have demonstrated that Lin28 blocks the processing of let-7 at primary, precursor, and mature forms of let-7 family members, as Microprocessor complexes (DGCR8 and Drosha) and DICER complexes cannot associate with Lin28-bound let-7 [15–17]. Furthermore, studies revealed that Lin28A and Lin28B inhibited let-7 biogenesis by distinct mechanisms. Lin28B sequesters primary let-7 transcripts and inhibits their processing by the Microprocessor [18], while Lin28A recruits TUTase4 to induce oligo-uridylation of pre-let-7, which blocks DICER processing and facilitates degradation of the RNAs [11–13]. Another study indicated that Lin28 used two different TUTases to control let-7 expression and had important implications for stem cell biology as well as cancer [19]. Taken together, regulation of let-7 expression is controlled by Lin28 proteins through the post-transcriptional blockade of let-7 biogenesis. Interestingly, Lin28A and Lin28B mRNAs themselves have potential let-7 complementary sites (3′UTR of Lin28) and serve as let-7 targets, making let-7 regulate Lin28 expression by cleaving Lin28 mRNAs or inhibiting translation of Lin28 mRNAs [6, 20–25]. Additionally, the let-7 target genes, such as c-MYC [26] and CDC25A [23], could regulate their downstream Lin28 *via* translational repression, making let-7 an indirect inhibitor of Lin28. Therefore, Lin28/let-7 axis establishes a double-negative feedback loop whereby either let-7 or Lin28 is expressed at high levels, promoting physiological or pathological conditions, respectively (Figure 2).

### Let-7 independent functionality

#### Interaction of Lin28 and mRNA

As for let-7 independent way, Cho *et al.* mapped the Lin28A binding sites on the genomic scale by RNA crosslinking-immunoprecipitation-sequencing (CLIP-seq) technology and ribosome footprinting. They observed that Lin28A binds to a large number of spliced mRNAs by recognizing AAGNNG, AAGNG, and less frequently UGUG, which are located in the terminal loop of a small hairpin [27]. In their study, Lin28A was found to be enriched in the peri-endoplasmic reticulum region and bound to the cytosolic surface of rough endoplasmic reticulum (RER) on which endoplasmic reticulum-associated mRNAs were translated in undifferentiated stem cells. Several other studies have reported that Lin28 could activate OCT4 mRNA translation through binding to OCT4 mRNA in complex with RNA helicase A in polysomes [28, 29], and that Lin28 could directly enhance translation efficiency of IGF2 mRNA during murine muscle cell differentiation [30]. It is worth mentioning that besides OCT4 and IGF2, researchers further found that a series of mRNAs bound by Lin28 were enriched in glycolysis and glucose metabolism, e.g., GPAA1 and GNPDA1 [31]. These interactions between Lin28 and mRNAs are based on linear sequence recognition motifs, Lei *et al*. also reported specific structural determinants for Lin28 binding with its targeted mRNAs, including HMGA1, EEF1G and RPS13 mRNAs [32]. More precisely, the motif is characterized by a small ‘A’ bulge flanked by two G:C base pairs embedded in a larger secondary structure, which mediates Lin28-dependent stimulation of mRNA translation.

#### Interaction of Lin28 and miRNA

**Figure 3:**
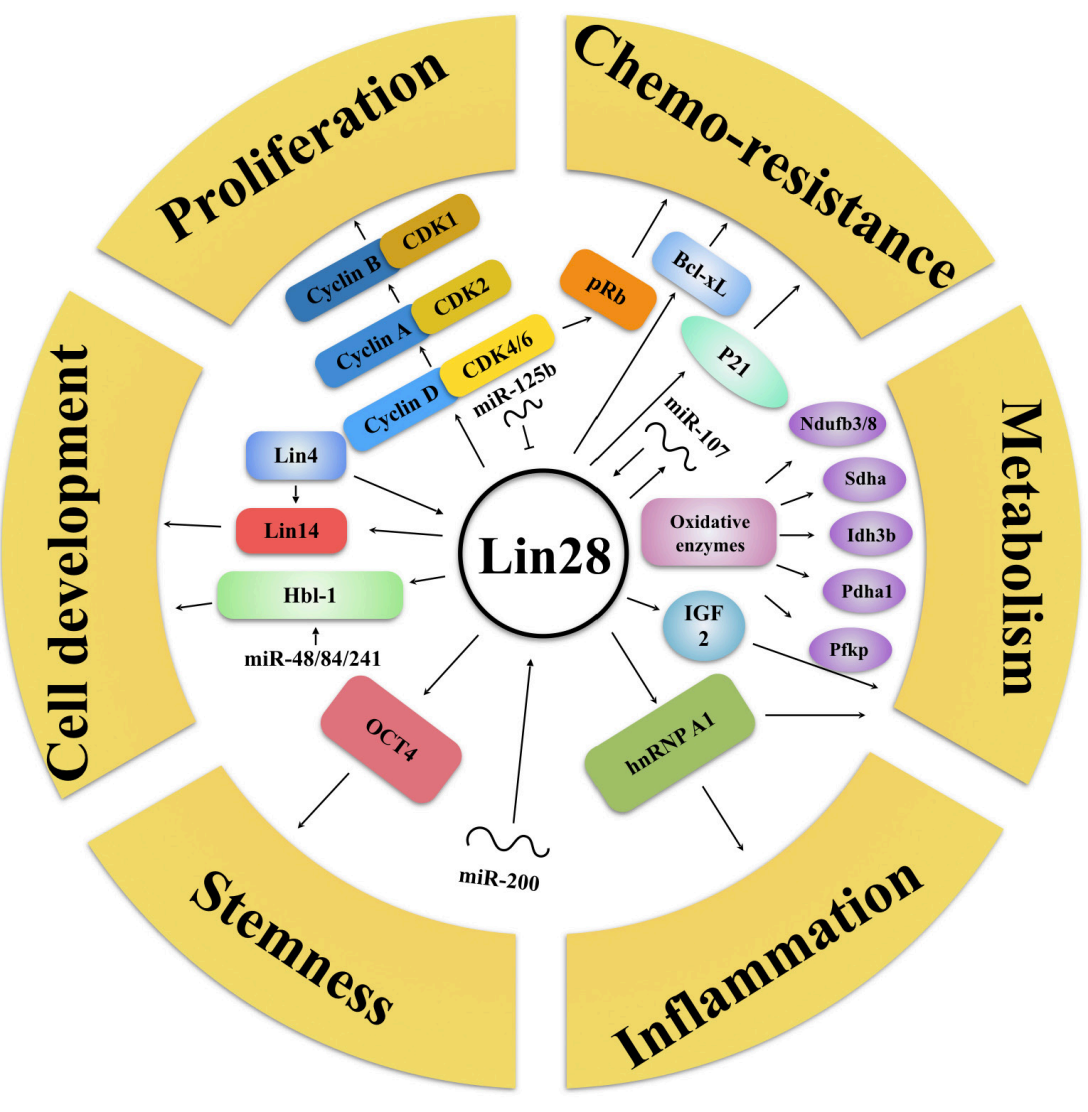
Lin28′s let-7-independent functionality Lin28 can regulate multiple tumor-associated progressions without let-7, but with proliferation (CyclinA/B/D, CDK1/2/4/6, miR-125b), chemoresistance (pRb, p21, Bcl-xL, miR-107), metabolism (IGF2, Oxidative enzymes), inflammation (hnRNP A1), stemness (OCT4, miR-200), cell development (Hbl-1, Lin4/14, miR-48/84/241) related proteins and RNAs. Arrows refer to promotion, while hammerheads refer to inhibition.

Lin28 also could activate or inhibit other miRNAs besides the let-7 family. For example, Lin28 inhibition has recently been demonstrated to decrease the level of abundance of miR-17∼92 family miRNAs, which may be connected with the molecular basis of GGAG motif in miRNAs [33]. Among aforementioned miRNAs, Peters *et al.* focused on the putative tumor suppressor miR-363, discovering that miR-363 RNA segment, which contains the GGAG motif, formed a relatively stable complex with the Lin28 protein. Knockdown of Lin28 could lead to decreased level of mature miR-363, implying that Lin28 functioned as a positive regulator of miR-363 biogenesis [34]. Additionally, Lin28 could differentially promote and inhibit specific fates of cells by inhibiting miR-302d with its CSD domain [35], miR-125b could efficiently reduce Lin28 protein levels *via* directly binding to the 3′-UTR region of the Lin28 mRNA [36]. Intriguingly, Willbert *et al.* have reported that Lin28 directly enhances its own expression level by binding to sites within its 3′UTR, revealing a mechanism of positive feedback regulation by Lin28 [37]. In fact, Lin28 can regulate multiple tumor-associated progressions in let-7 independent way, including proliferation, chemo-resistance, metabolism, inflammation, stemness and cell development (Figure 3).

## MULTIPLE PROCESSES REGULATED BY LIN28 IN BREAST CANCER

**Figure 4:**
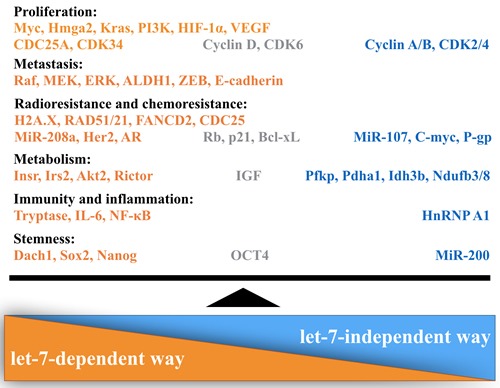
Lin28 regulates multiple progressions in breast cancer Lin28 exerts its critical role in breast cancer through two distinct ways: let-7 dependent and let-7 independent. Orange refers to factors of let-7-dependent way, blue refers to factors of let-7-independent way, gray means factors involved in both mechanisms.

In malignancies, a more common phenomenon is that the expression of Lin28 is up-regulated. The correlational studies have shown that Lin28 may be transcriptionally activated by upstream factors, e.g. c-Myc, Src, NF-κB and Wnt [18, 38]. For instance, c-Myc is found to be associated with the genomic locus encoding Lin28 and transactivate the Lin28 promoter, leading Lin28 to become the direct target of MYC [39]. Besides, Chen *et al*. demonstrated that the C allele of rs3811463, a SNP that located near the let-7 binding-site of Lin28, could weaken the suppression of Lin28 by let-7, which means an increasing level in Lin28 expression along with a reduction of let-7 level, elevating the risk of breast cancer [40]. In fact, Lin28 is reactivated by oncogenes in approximately 15% of all analyzed cancers, and its expression is correlated to advanced stages of various types of cancer, including breast cancer [41]. Using tissue microarrays, researchers have demonstrated that Lin28 is expressed in a collection of human breast cancer cell lines, while cultured normal human breast epithelial cells have no detectable Lin28 expression. Furthermore, Lin28 expression was detected in ductal carcinoma *in situ* (DCIS) specimens but not in any benign breast tumor specimens [42]. Taken together, these data have shown that expression of Lin28 is frequently up-regulated in breast cancer. Here, we will overview mechanisms and functions of Lin28 as well as multiple cancer-associating progressions regulated by Lin28 (Figure 4).

### Lin28 promotes cancer cell proliferation

Arguably, the most fundamental feature of Lin28 in breast cancer cells is it ability to promote and sustain chronic proliferation. Many studies focusing on proliferative signaling in cancers suggest that Lin28 functions as an oncogene by repressing let-7, leading to the dysregulation of multiple genes regulated directly or indirectly by let-7, including MYC, Hmga2, and components of PI3K-mTOR pathway [43–46]. In the aspect of cell cycle, Li *et al.* have demonstrated that Lin28 promotes proliferation of tumor cells through regulating the G0/G1 transition in cell cycle, namely, increasing the expression of Cyclin D1/D2, CDC25A, CDK34, CDK6, as well as other cell cycle-related factors by depressing let-7 [42]. We should note that Figure 5 in Li's article showed that Lin28A dramatically promoted the G0/G1 transition rather than its role of enhancing G2/M transition in human embryonic stem cells (ESCs), further works are needed to explain the reason why Lin28A controls different cell cycle regulators among different cells. Adding another layer of meaning to the complex downstream effects of Lin28 are some studies in which Lin28 itself binds to many mRNAs and enhances their translation, including IGF2 and Cyclin A/B [30, 35].

Furthermore, Sharma *et al.* reported that combining the Lin28/let-7a/Kras axis inhibitors (NVP-LDE-225 and NVP-BEZ-235) could inhibit tumor growth by inhibiting Bcl-2 family members and activating caspases and by suppressing PI3K-mTOR pathway, suggesting that Lin28/let-7a may be involved in the apoptosis and proliferation of cancer cells simultaneously [47]. Since several studies indicate that the tumor-associated neovasculature, generated by the process of angiogenesis, could also promote tumor growth, researchers have investigated in the involvement of Lin28/let-7 axis in angiogenesis. For instance, Isanejad *et al.* found that markers for aggressive breast cancer cells (such as Ki67 and ERα) or for tumor blood vessels (such as HIF-1α, CD31 and VEGF) could be down-regulated by the combination hormone therapy through the let-7a pathway. Taken together, these evidences support the hypothesis of Lin28/let-7 axis contributing to the anti-angiogenesis effects of the breast cancer [48].

### Lin28 expression promotes cancer cell metastasis

Abnormal Lin28 activation is found in diverse human malignancies, especially in poorly differentiated and highly aggressive tumors [49]. Rosner *et al.* have revealed that Lin28 facilitates bone metastasis in Raf kinase inhibitory protein (RKIP)-expressing breast tumors [50]. More specifically, the results support a model in which RKIP suppresses Raf-1/MEK/ERK activity, leading to the inhibition of Lin28 and induction of let-7, finally blocking the induction of Snail transcription and other genes involved in tumor cell invasion and metastatic colonization. Epithelial-to-mesenchymal transition (EMT) is known to accelerate tissue remodeling from epithelial phenotype to mesenchymal phenotype, and Lin28/let-7 axis is also a prerequisite for the process of EMT among some cases [51]. Liu *et al.* found that over-expression of Lin28 in breast cancer cells remarkably decreased the expression of E-cadherin but increased Vimentin, thus facilitating metastasis [52]. In the Table 3 of the article, they also reported that Lin28 correlated with axillary lymph node, distal metastases and PR. However, the authors found no significant correlation between Lin28 and ER or HER2 expression, therefore large-scale clinical samples are needed to ensure Lin28 to serve as a prognostic marker for patients with breast cancer. Furthermore, Wang *et al.* demonstrated that in breast cancer cell lines, let-7a could suppress cell migration by significantly blocking the direct binding target of Lin28, which provided evidence for the potential therapeutic role of targeting Lin28 strategies in conquering metastasis in breast cancer [53].

### Lin28 contributes to the resistance of radiation treatment and chemotherapy

Resistance to radiotherapy and chemotherapy has long been a great challenge in the treatment of cancer patients, and abundant evidences have shown that over-expression of Lin28 contributed to cancer cell radiotherapy and chemotherapy resistance [54]. For instance, Wang *et al.* found that Lin28 expression was up-regulated in radiation-resistant breast cancer cells. Lin28 transfection could induce radiation resistance *via* inhibiting H2A.X pathway while over-expression of let-7 enhanced the sensitivity to radiation [55]. Sun *et al.* have demonstrated that miR-208a-SOX2/β-catenin-Lin28-let-7a-DICER1 regulatory feedback loop participates in the therapy resistance of breast cancer through promoting the induction of the cancer stem cells [56]. Based on the Figure 5 of their work, miR-208a increased the activity of DICER1 while let-7a directly targeted and degraded DICER1 mRNA, it is necessary to investigate whether the observation could be explained by the competitive endogenous RNA (ceRNA) hypothesis. Since the impact of Lin28/let-7 axis on radio-sensitivity has been confirmed *in vitro*, increasing evidence has shown that down-expression of Lin28 and over-expression of let-7 could decrease the expression of RAS oncogene and genes associated with DNA like RAD51, RAD21, FANCD2 and CDC25, eventually radiosensitizing the cancer cells [57–59].

In the aspect of drug resistance, Teng *et al.* reported that over-expression of Lin28 decreased the sensitivity to chemotherapy (e.g. Oxaliplatin, Paclitaxel, Doxorubicin and Fluorouracil) *via* inhibiting miR-107, as well as the RNA and protein expression of c-Myc and P-gp [60]. Other researchers also have found that Lin28-induced chemotherapy resistance is associated with let-7, Rb, p21 and Bcl-xL, thus unraveling complicated relationship between Lin28 and tumor resistance [61, 62]. As for clinical study, by evaluating the polymorphism of Lin28 gene between breast cancer females and healthy controls, researchers have found that genetic variations in Lin28 might be a possible mechanism underlying susceptibility and clinical features in breast cancer, including the response to neoadjuvant chemotherapy [63].

### Lin28 contributes to enhanced cell metabolism

Sixty years ago, Otto Warburg first noted that under normoxic conditions, normal cells metabolize glucose by using mitochondrial oxidative phosphorylations (OxPhos) instead of glycolysis to maximize the production of adenosine triphosphate (ATP), but cancer cells choose to rely on glycolysis even under the sufficient oxygen condition, a phenomenon later known as the Warburg effect [64]. Since then interest in this topic has increased, and major areas of knowledge have been gained, but fundamentally important questions remain unresolved.

Extensive studies have provided the evidence that conditional deletion of Lin28 could lead to insulin resistance and impaired glucose uptake. Let-7 transgenic mice not only reduced body size and growth retardation, but also led to hyperglycemia and glucose intolerance [45]. As for breast cancer, Yang *et al.* performed RNA-protein immunoprecipitation (RIP) coupled with genome-wide sequencing (RIP-Seq) to identify endogenous Lin28 mRNA targets. Data suggested that Lin28 regulated the expression of a unique set of mRNAs, whose expression is involved in classic cancer metabolism [65]. More accurately, Lin28 down-expression could inhibit insulin sensitivity mostly by suppressing the let-7 targets Insr, IGF1r, Irs2, Akt2, and Rictor, and also in part by directly reducing the ribosomal translation of mRNAs encoding IGF2 and mitochondrial OxPhos enzymes.

Of course, other researches on metabolism have different voices. Zhang *et al.* found that induction of Lin28 over-expressing mouse ESCs compromised basal and maximal oxygen consumption rate (OCR), but that levels of let-7 did not change from their already suppressed levels [66]. According to Figure S4 in their study, authors not only verified that let-7 mimic in Lin28-overexpressing cells could not reverse the reduced OCR, but also knockdowned Lin28 in Dgcr8^−/−^ ESCs (scant quantities of let-7) to exclude other possibly let-7-related microRNAs function. Both the experimental design and result are quite rigorous and precise, which deserves our respect and learning. Other studies also found that gain or loss of function of let-7 in wild-type cells did not change OCR, suggesting that Lin28 reduced OCR through several let-7-independent mechanisms [67].

### Lin28 is involved in immunity and inflammation

In addition to the above-mentioned role of Lin28 in cancer, several frontier researches have suggested that Lin28 may be implicated in the immune system [68]. For example, researchers reported that Lin28 in myeloid could affect mast cell differentiation and promote mast cell malignancy [69]. Mast cells, the critical components of the innate immune system, release a serine protease called Tryptase, which was demonstrated to play a positive role in tumor angiogenesis of breast cancer [70]. Several other investigators have provided evidence that Lin28 promotes tumor growth *via* enhancing the development of T cells, B cells, and natural killer T (NKT) cells [71–74].

In regards to inflammation, Iliopoulos *et al.* have observed that Src, an oncoprotein in immortalized breast cells, could activate a rapid inflammatory response mediated by NF-κB. The response then led to Lin28-regulated expression of the anti-inflammatory cytokine interleukin-6 (IL-6) *via* inhibiting let-7 expression, thus revealing a new mechanism containing cancer cells and immune molecules [75, 76]. They also found that let-7 inhibited IL-6 expression both directly through its 3′UTR and indirectly by interacting with RAS to reduce the NF-κB activity. As for other inflammatory mechanisms, Yang *et al.* have reported that breast cancer cells lacking Lin28 could increase levels of anti-inflammatory cytokines, and that the regulation of the major cytokine genes is dependent on the expression of hnRNP A1, suggesting a mechanism independent of let-7 [65]. Meanwhile, Yang *et al.* also found that despite of the cooperation to regulate nuclear processes, depletion of Lin28 and hnRNP A1 proteins yielded nonidentical effects on mRNA splicing (presented in Figure 6 of the article). The phenomenon may be attributed to that RT-PCR assay is not sensitive enough and transcript isoforms are confounded by other cellular factors.

### Lin28 is involved in cancer stem cells

In the cancer microenvironment, a fraction of tumor cells, called tumor initiating cells (TICs) or cancer stem cells (CSCs), have been found to propagate in various tumors, including breast cancer [77]. Human mammary epithelial cells transformed by EMT could significantly enhance their self-renewal and tumor-initiating capabilities, and lead to the expression of stem-cell markers typically associated with the stemness of breast cancer cells [78]. Studies in the past decade have shown that the Lin28/let-7 axis plays a significant role in stem cell renewal [79]. For example, Cai *et al.* have reported that Lin28 upregulation and let-7 posttranscriptional downregulation were identified in the Wnt-β-catenin pathway-stimulated breast CSCs phenotype, while loss of function of Lin28 impaired Wnt-β-catenin-pathway-mediated let-7 inhibition and breast cancer stem cell expansion [80]. The in-depth mechanism revealed that Lin28 could act as a novel direct downstream target of the Wnt-β-catenin pathway, namely, both LEF1 and β-catenin were found to directly bind to the Lin28 promoter. Feng *et al.* have implicated that over-expression of HER2 in breast cancer increases the population of CSCs, which also correlates with the high Lin28 expression level [81]. Specifically, their discoveries suggested that Lin28 could bind to HER2 mRNA and stimulate its translation *via* a 200-nucleotide sized Lin28-responsive element (LRE) in HER2 mRNA. But according to the Figure 4 illustrated in Feng's article, targeting HER2 alone by Herceptin (Trastuzumab) is insufficient to inhibit the level of Lin28, thus the study of Lin28/HER2 positive feedback loop still need to be investigated. Moreover, Wu *et al.* found that cell fate determination factor Dach1 reduced breast tumor formation in serial transplantations *in vivo*, further unraveling that in breast cancer cells Dach1 directly bonds to the promoter region of Lin28, Sox2 and Nanog genes and inhibits their expression, finally decreases expansion of CSCs population and blocks breast tumor growth [82]. It is worth mentioning that Jolly *et al.* discovered that independent of let-7, Lin28 was strongly inhibited by miR-200 which pushed epithelial end towards mesenchymal end of CSCs, thus allowing mesenchymal phenotype cells to gain stemness [83] (Table 1).

**Table 1:** Multiple processes regulated by Lin28 in breast cancer

Tumorigenic mechanisms	Key detection methods	Major findings	References
Proliferation	Immunocytochemistry, Microarray analysis, Motility assay	Lin28 promoted cell proliferative signaling like PI3K-AKT-mTOR pathway and its target genes, such as Myc, Hmga2. Lin28 promoted the G0/G1 transition of cell cycle by regulating cell cycle-related factors, such as Cyclin D1 and D2, CDK6. Lin28 inhibited tumor growth by modulating Bcl-2 family members and activating caspases. Lin28 was involved in the anti-angiogenesis effect of the breast cancer.	[30, 35, 42–48]
Metastasis	Transwell migration assay, Flow cytometry analysis	The inhibition of Lin28 blocked the expression of genes involved in tumor cell invasion and metastatic colonization. Lin28 facilitated breast cancer metastasis by promoting EMT.	[49–53]
Radiotherapy and chemotherapy resistance	Immunofluorescence,Cell drug-resistance assay, Xenograft Assay	Lin28 transfection induced radiation resistance via inhibiting H2A.X pathway. Lin28 increased radioresistance by affecting the RAS oncogene and genes associated with DNA damage repair. Lin28 decreased chemosensitivity via inhibiting miRNA-107, let-7, Rb, p21 and Bcl-xL. Lin28 was associated with susceptibility and clinical features in breast cancer, including the response to neo-adjuvant chemotherapy.	[54–63]
Metabolism	Immunoprecipitation, RIP-Seq, Metabolomics Analysis	Lin28 regulated the expression of a unique set of mRNAs involved in cell metabolism. Lin28 downexpression could inhibit insulin sensitivity mostly by regulating the let-7 targets Insr, Igf1r, Irs2, Akt2, and Rictor. Lin28 overexpression compromised basal and maximal oxygen consumption rate (OCR).	[45, 64–67]
Immunity and inflammation	Flow cytometric analysis, RNA-Seq, Immunoprecipitation,ELISA	Lin28 could affect mast cell differentiation and promote the mast cell malignancy. Lin28 promoted tumor growth via enhancing the development of T cells, B cells, and natural killer T (NKT). Lin28 regulated the expression of the anti-inflammatory cytokine interleukin-6 (IL-6) via inhibiting let-7 or depending on hnRNP A1 expression.	[65, 68–76]
Stemness	Microarrays, Immunoprecipitation, In vivo stem cell assay	Loss of function of Lin28 impaired breast cancer stem cell expansion through Wnt-β-catenin-pathway. Lin28 increased the population of cancer stem cells (CSCs) by activating HER2 expression or cell fate determination factor DACH1. Lin28 could be strongly inhibited by miR-200 and allowed CSCs to gain stemness.	[77–83]

## CURRENT OPINIONS AND FUTURE PERSPECTIVES ON LIN28 IN BREAST CANCER

Breast cancer is a disease plagued by recurrences with progressive drug resistance, ultimately leading to uncontrolled cancer progress. It is now evident that Lin28 does play roles in breast cancer initiation, progression and maintenance. However, the challenge that lies ahead is how to integrate our improved molecular understanding of Lin28 and breast cancer pathogenesis with novel therapeutic options, ultimately improving patient outcomes.

The canonical targets of Lin28, let-7 family members, have been most notably implicated in cancer [84]. The association of let-7 with pathogenesis of breast cancer is supported by studies examining let-7 expression in breast cancer cell lines and clinical samples (Table 2). The majority of studies revealed that most of let-7 family members were down-regulated in breast cancer samples with either lymph node metastasis or higher proliferation index *versus* normal tissues [85–88], while one study showing up-regulation of let-7b [89]. Subsequently, several functional studies have reported novel mechanisms of let-7 in breast cancer cells [90–97]. As for clinical researches, apart from cases for let-7 as the classical suppressor [98], other studies have suggested that let-7 does not function as a tumor suppressor under all circumstances [99–101].

**Table 2:** The expression of let-7 in breast cancer

let-7 family members	Down-regulated	Up-regulated	Targets	Reference
let-7a-1	√			[85]
let-7 family members	√			[86]
let-7a-2/let-7a-3/let-7d/let-7f-2/let-7i	√			[87]
let-7a-2	√			[88]
let-7b		√		[89]
let-7d/let-7f/let-7a/let-7e/let-7c/ let-7g/let-7i	√			
let-7a	√		H-RAS, HMGA2	[90, 91]
let-7a	√		CCR7	[92]
let-7d	√		Wnt	[93]
let-7c	√		Wnt	[94]
let-7 family members	√		Critical components of ER/mitogenic/cell cycle signaling pathways	[95]
let-7b	√		PAK1, DIAPH2, RDX	[96]
let-7b	√		BSG	[97]
let-7a/let-7b/let-7c/let-7i/let-7g	√		GAB2, FN1	[98]
let-7a-2/miR-98		√	ADRB2*,CEP164*,CYP19A1*,TARBP2*	[99]
let-7i	√			[100]
let-7i		√		[101]

*Predicted putative targets

For neoteric findings, studies using 26 breast cancer surgical specimens found Lin28 expression to be positively correlated with ER and PR status but inversely correlated with HER2 status [102, 103]. In addition to the known risk factors like ER, PR and HER2, Shen *et al.* have found that Lin28 and AR (androgen receptor) co-expression is a strong predictor of poor prognosis in breast cancer [104]. Although the positive feedback mechanism of Lin28 and AR is still not clear, researchers can conduct in-depth studies in this new research domain. Moreover, Liu *et al.* found that Hepatitis B X-interacting protein (HBXIP), a novel oncoprotein, could promote proliferation of breast cancer cells by up-regulating Lin28B [105]. Segalla *et al.* reported that the ribonuclease DIS3 could promote let-7 maturation and inhibit Lin28B mRNA levels through recognition of AU-rich elements in the 3′UTR [106]. Therefore, therapeutically canonical and non-canonical key factors may serve as the target to control upstream or downstream miRNAs and direct mRNA regulation of Lin28, which will guide attempts to use Lin28 as a target for treating human breast cancer.

Recent advanced studies also have revealed that long non-coding RNA H19 acts as a sponge to antagonize let-7. For example, the ability of let-7 to repress the expression of an array of metastasis-promoting genes is compromised when H19 expression is high, leading to decreased bio-availability of let-7, increased expression of c-Myc, Hmga2 and Imp3, and activation of cell migration and invasion [107], thus prompting us to study the relationship among H19, let-7 and Lin28 during carcinogenesis. Researchers’ findings also included that metformin, a drug used for treating type-2 diabetes, could down-regulate H19 at least in part by altering methylation of the H19 promoter, thus presenting anti-tumor effects on tumor cells. In addition, Lin28 has been found to participate in other popular research fields, including exosomes, autophagy and cells polarity. For instance, high Lin28A expressing exosomes could induce EMT-related gene expression and promote non-metastatic target cells to migrate and invade [108], Lin28 suppressor let-7 could activate autophagy by repressing the mTOR signaling pathway [109], human induced pluripotent stem cells (iPSCs) generated by Lin28 could spontaneously differentiate into polarized retinal pigmented epithelium [110]. Taken together, it is necessary for us to further investigate the concrete mechanism of Lin28, which may open up new research avenues for cancer diagnostics and treatment.

In fact, aberrant expression of Lin28 stimulates tumorigenesis in many other kinds of cancers, including lung [111], hepatocellular [112], gastric [113], colon [114], kidney [115], and ovarian carcinoma [116]. More precisely, novel findings involve that Lin28 interacts with a protein expressed by Hepatitis B virus to enhance the proliferation of hepatoma cells [112], Lin28 activates genes that simultaneously enhance both metastasis and cell differentiation in colon cancer [114], higher Lin28 expression is associated with worse pathologic tumor responses under the neoadjuvant chemotherapy [113], Lin28 expression in kidney cells promotes metabolic switching (the increasing rate of glycolysis) to a phenotype related with cancer [115]. IL-1β-miR-101-Lin28 is certified as a novel pathogenic inflammatory signaling in non-small cell lung cancer [111]. Lin28 together with OCT4 can be used to identify subgroups of stem cell-like cells in ovarian cancer [116]. Additionally, except for researches that Lin28/let-7 loop is involved in ten hallmarks of cancer [117], the aberrant loop also regulates cellular senescence and has connection with various oncogenes and signaling pathways, including MYC, RAS, MAPK signaling and PI3K/AKT signaling [118]. Although above mechanisms have not fully been verified in breast cancer, they could provide us new thoughts and references, based on similarity of traits of Lin28 and relative factors, we may propose a series of bold experiments to promote new discoveries.

Nowadays, available data suggest that inhibition of Lin28 in breast cancer holds great therapeutic potential for the treatment of breast cancer. However, it remains to be determined when and to what extent, Lin28 might be involved in preventive, predictive or prognostic functions. Further endeavors will unravel its precise roles in the pathogenesis of breast cancer. It is not difficult to predict that exciting times are still ahead for researches studying Lin28.

## CONCLUSIONS

In summary, Lin28 has been observed to be frequently up-regulated in breast cancer. Through canonical and non-canonical pathways, high levels of Lin28 could promote cellular proliferation, metastasis, radio- and chemo-resistance, metabolism reprogramming, immunity and inflammation, and tumor-associated stemness in breast cancer. Although the Lin28 related regulatory network remains unclear, it would certainly be an intriguing frontier to study and a valuable hotspot for future breast cancer therapeutic strategies.
